# A Resilience-Based Intervention to Mitigate the Effect of HIV-Related Stigma: Protocol for a Stepped Wedge Cluster Randomized Trial

**DOI:** 10.3389/fpubh.2022.857635

**Published:** 2022-03-29

**Authors:** Xiaoming Li, Shan Qiao, Xueying Yang, Sayward E. Harrison, Cheuk Chi Tam, Zhiyong Shen, Yuejiao Zhou

**Affiliations:** ^1^South Carolina SmartState Center for Healthcare Quality, Arnold School of Public Health, University of South Carolina, Columbia, SC, United States; ^2^Department of Health Promotion, Education, and Behavior, Arnold School of Public Health, University of South Carolina, Columbia, SC, United States; ^3^Department of Psychology, College of Arts and Sciences, University of South Carolina, Columbia, SC, United States; ^4^Guangxi Zhuang Autonomous Region Center for Disease Prevention and Control, Guangxi, China

**Keywords:** HIV, stigma, resilience, intervention, stepped wedge cluster randomized trial, viral suppression

## Abstract

**Background:**

Despite decades of global efforts to tackle HIV-related stigma, previous interventions designed to reduce stigma have had limited effects that were typically in the small- to-moderate range. The knowledge gaps and challenges for combating HIV-related stigma are rooted both in the complexity of the stigma and in the limitations of current conceptualizations of stigma reduction efforts. Recent research has shown the promise of resilience-based approaches that focus on the development of strengths, competencies, resources, and capacities of people living with HIV (PLWH) and their key supporting systems (e.g., family members and healthcare providers) to prevent, reduce, and mitigate the negative effects of stigma. However, the resilience-based approach, while hypothesized, has rarely been empirically tested in large intervention trials, especially in resource-limited settings.

**Methods:**

In this study, we propose to develop, implement, and evaluate a theory-guided, multilevel, multimodal resilience-based intervention *via* a stepped wedge cluster randomized trial among 800 PLWH and their biological or surrogate family members, as well as 320 healthcare providers in Guangxi, China with a longitudinal follow-up period of 36 months at 6-month intervals. The primary outcome will be viral suppression and the intermediate outcomes will include perceived stress and medication adherence of PLWH as well as resilience measures at the level of the individual, the family, and the healthcare system.

**Discussion:**

The proposed study will be one of the first large scale efforts to examine whether resilience among PLWH can be fostered and sustained through a multilevel and multi-component HIV-related stigma intervention and whether a resilience-based intervention can improve clinical outcomes and quality of HIV care among PLWH in a low-resource setting. If efficacious, the intervention components could be tailored to other groups of PLWH and adapted for other low- and middle-income countries.

**Trial Registration:**

This trial is registered at ClinicalTrials.gov, registration number NCT05174936, registered 13 December 2021. https://register.clinicaltrials.gov/prs/app/action/LoginUser?ts=3&cx=-jg9qo2.

## Introduction

The finding from the HPTN 052 trial of a 96% reduction in HIV incidence among discordant couples when the HIV-positive partner receives antiretroviral therapy (ART) has led to the emergence of “treatment as prevention” as the dominant strategy to end the global HIV epidemic (1–3). However, numerous obstacles continue to prevent appropriate treatment and optimal clinical outcomes, including stigma against people living with HIV (PLWH). This is a significant public health problem worldwide, particularly in low- and middle-income countries (LMICs), including China. Stigma and discrimination related to HIV and AIDS (i.e., “HIV-related stigma”) can be multifaceted (e.g., individual, community, and institutional) and lead to detrimental impacts, such as preventing PLWH from seeking and receiving appropriate treatment and care, contributing to psychiatric disorders (e.g., depression) and low quality of life, and producing poor clinical outcomes among PLWH (4–6).

Despite substantial global efforts to reduce HIV-related stigma, stigma and discrimination remain persistent and widespread and are among the most poorly understood aspects of the epidemic (7). Two earlier systematic reviews of HIV-related stigma reduction interventions conducted in almost 30 countries across a time span of 20 years (8, 9) identified numerous gaps in evidence-based interventions to reduce HIV-related stigma. These gaps included insufficient engagement of PLWH, only targeting a single socioecological level or a single domain of stigma, inadequate measures to evaluate HIV-related stigma reduction, limited public health relevance of the findings (e.g., lack of clinical endpoints), and a lack of rigor in research methodology. Likewise, Rao et al. in their 2019 review (10) of 24 multilevel stigma interventions around the globe confirmed that the effects of these interventions varied widely in magnitude and were typically in the small-to-moderate range. The authors acknowledged that while there has been progress over the past decade in developing and evaluating multilevel stigma interventions, much work remains to strengthen and expand this approach. A collection of studies in the 2020 *AIDS* special issue titled “Reducing stigma and discrimination: innovation in measurement and practice” also suggested that HIV-related stigma remains highly relevant and persistent in multiple cultural settings and the gains from HIV-related stigma reduction initiatives have often been modest and rarely implemented at scale (7).

Many of the existing knowledge gaps and challenges for combating HIV-related stigma are rooted in the complexity of the stigma and in limitations in current conceptualizations of stigma reduction efforts (11–13). The focus of most existing intervention research on HIV-related stigma has primarily targeted HIV-negative populations with a goal of eradicating or reducing HIV-related stigma against PLWH and their families. However, HIV-related stigma, like other types of social stigma, has proven difficult to eradicate at the population level. This suggests that, in addition to continued efforts to decrease HIV-related stigma globally, interventions are also needed to help individuals targeted by stigma and discrimination, such as PLWH, to cope with stigma-related challenges (e.g., negative interpersonal experiences, stress) (1, 12). As such, based on their HIV Disparities Model and evidence from the global literature, Earnshaw and colleagues proposed a resilience agenda and suggested “enhancing resilience to societal stigma at the individual (e.g., PLWH, family members) and structural levels (e.g., healthcare settings) as a critical strategy to reduce HIV disparities” (page 231) (3). Recent multi-country analyses of data from the PLWH Stigma Index 2.0 also suggest that multilevel interventions are a promising approach to promote resilience among PLWH and to support PLWH engaging in services, adhering to ART, achieving and maintaining viral suppression, and achieving high quality of life (14, 15).

Resilience in the face of adversity or stress is a necessary capacity of human development (16, 17). Over the past three decades, the definition of resilience has evolved from the view of resilience as an individual personality trait (18), to an ecological view that resilience is a dynamic, multidimensional construct that incorporates bidirectional interaction between individuals and their environment (e.g., family, peers, community, society) (6, 18). Resilience can be cultivated or enhanced in an individual's life by the presence of one or more protective factors, such as close relationships with competent and caring partners in family and healthcare settings, a range of problem-solving skills, and a variety of psychological dispositions (e.g., self-esteem, self-confidence, positive future orientation) (9–20). Available data suggest the promises of these protective factors to foster resilience and mitigate the negative effect of stigma on HIV clinical outcomes (21, 22). The global literature has suggested the critical role of family members [either of origin (e.g., parents, spouse, siblings) or of choice (e.g., non-kin individuals who serve a family-like role by providing emotional or other support)] (23, 24) as a “network of mutual commitment” in assisting PLWH in achieving viral suppression and coping with HIV-related stigma (25, 26). The role of supportive family members in coping with HIV-related stigma appears especially salient in collectivistic societies such as China, where socio-cultural norms emphasizing the individual's obligation and responsibility to the family and community may indirectly facilitate the manifestation of HIV-related stigma. These findings indicate that interventions for HIV-related stigma can benefit from taking into account culturally appropriate intra- and interpersonal protective factors and seeking to actively enhance these resilience factors.

Healthcare providers (HCPs) can also play an essential role in promoting resilience against HIV-related stigma. While healthcare systems in China and other LMICs are facing increasing demands to provide quality care to a growing population of PLWH, healthcare settings are often the places where PLWH experience stigma and discrimination (27, 28). Because of the critical importance of engaging in HIV care for PLWH's quality of life and clinical outcomes, global literature has suggested the value of supportive healthcare systems, including HCPs, across various HIV epidemics and socio-cultural settings (28, 29). Previous work in both China (30–32) and other countries (33–38) has shown that interventions among HCPs can reduce HIV-related stigma by improving their knowledge about the prevention and treatment of HIV and AIDS, as well as their willingness to treat and support PLWH, which can lead to better quality of care for PLWH. Recent research, including our own preliminary data (39–43), has suggested the potential utility of adopting a resilience-based approach that focuses on the development of strengths, competencies, resources, and capacities in PLWH, as well as their families and healthcare systems to reduce and actively mitigate the negative effects of stigma. However, this approach, while hypothesized, has not been sufficiently tested in longitudinal studies or large intervention trials, especially in low-resource settings.

## Conceptual Framework

**Figure 1:**
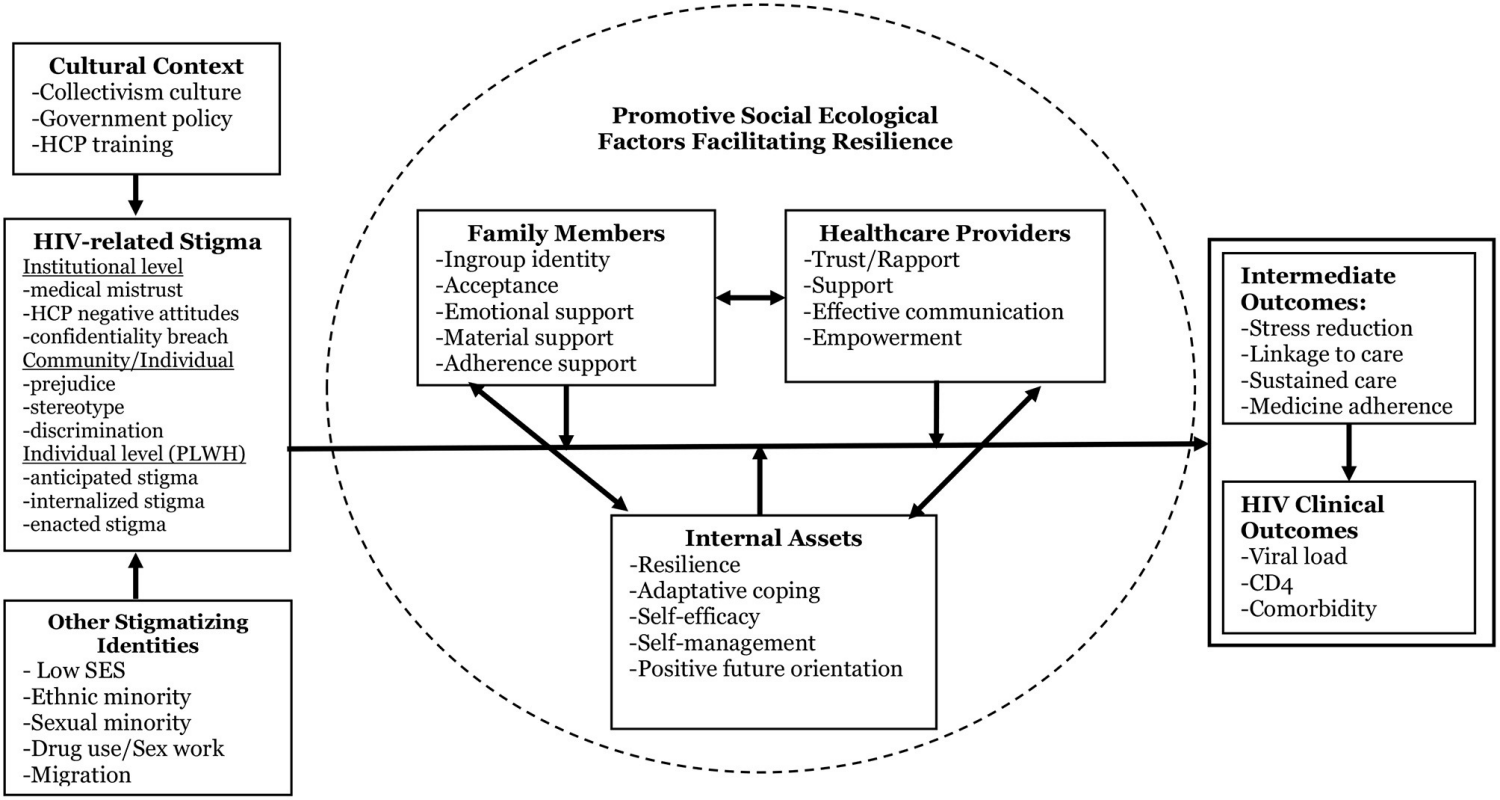
Conceptual framework [adapted from Li et al. (20)].

Figure 1 presents a multi-component, multi-level conceptual framework we developed that depicts the process of HIV stigma manifestations and role of resilience development in mitigating the negative effects of stigma on psychological, behavioral, and clinical outcomes. This framework is culturally adapted from an existing resilience-based intervention conceptual framework (20) by (1) specifically targeting PLWH and two key supporting sources in their lives, and (2) using HIV-related clinical outcomes as endpoint measures. Guided by the core principles of the socio-ecological model of human development (44), positive psychology, and theories of resilience (45), this model focuses on the dynamic process of resilience within the cultural context of multi-layered HIV stigma and emphasizes the centrality of individual assets and supportive social contexts in resilience development (46). Support from family members and HCPs, two critical entities in the context of HIV care and HIV-related stigma, can foster the development of PLWH's internal assets (e.g., self-esteem, self-efficacy, positive future orientation). A positive impact of the proposed intervention on supportive social contexts and PLWH's internal assets will in turn develop resilience and produce improvement in intermediate outcomes (e.g., stress, medical adherence) and endpoint clinical outcomes among PLWH. The framework emphasizes individual and contextual factors (including diverse family structures and risk profiles) that may mediate or moderate the effects of the proposed intervention (13, 27). The framework also provides a “blueprint” that guides our measures for each of these critical domains and our analytic plan to test intervention effects and potential individual and contextual mediators and moderators of such effects.

## Aims and Objectives

This study proposes to develop, implement, and evaluate a theory-guided, multilevel, multimodal resilience-based intervention *via* a stepped wedge cluster randomized trial among 800 PLWH and their biological or surrogate family members, as well as among 320 healthcare providers in Guangxi Zhuang Autonomous Region (“Guangxi”), China with a longitudinal follow-up over a period of 36 months with 6-month follow-up intervals. The specific aims are to:

(1) Develop a multilevel **r**esilience-based H**I**V **s**tigma r**e**d**u**ction and mitigation intervention **p**rogram (“RISE-UP”) engaging PLWH, their biological or surrogate family members, and healthcare providers (HCPs). The RISE-UP intervention will target individual factors (e.g., resilience, self-efficacy), family factors (e.g., supportive family members), and structural factors (e.g., supportive HCPs and care facilities) by adapting existing intervention components that have shown preliminary efficacy in China or elsewhere.
(2) Test the short-, medium-, and long-term efficacy of the RISE-UP intervention through a stepped wedge cluster randomized trial among 800 PLWH-family member dyads and 320 HCPs from ~40 HIV clinics in Guangxi with a longitudinal follow-up over a period of 36 months at 6-month intervals.
(3) Identify individual and contextual factors that may mediate or moderate the effect of RISE-UP intervention on viral suppression, other clinical outcomes (e.g., CD4), and the intermediate psychosocial and behavioral outcomes (e.g., resilience, stress, medication adherence, quality of life).

## Methods/Design

### Population and Setting

#### Research Setting

This proposed study will be conducted in Guangxi, one of the regions in China that is experiencing the fastest growth of the HIV epidemic. A total of 10,060 new HIV cases were reported in Guangxi in 2020 with a male to female ratio of 2.66:1. The HIV epidemic in Guangxi is characterized by: (1) sexual transmission (97.8% of all cases, including 90.5% heterosexual transmission and 7.3% male-to-male sexual transmission); (2) rural residents (72.6%); (3) low education levels (84.9% ≤ middle school education, including 7.3% with no formal education, 44.6% with elementary school education only, and 33.1% with middle school education only); and (4) late diagnosis (52.0% of newly reported cases were AIDS patients). More than half (54.1%) of the cases were married, followed by divorced or widowed (21.4%). Individuals ≥50 years of age accounted for 51.0% of all the cases living with HIV.

#### Selection of Participating Sites

In Guangxi, geographic units important for our study design include cities that are composed of urban districts, as well as rural counties that are composed of rural townships. There is one designated primary public hospital with an HIV clinic in each urban district and rural township that works under the direction of the city or rural county Center for Disease Control and Prevention (CDC) to conduct clinical management and semi-annual follow-ups for all PLWH in the district or township. In collaboration with Guangxi CDC, we will select two cities and eight rural counties that have the largest number of reported HIV cases to participate in our proposed study. Guangxi CDC will identify all urban districts in the two cities and all rural townships in the eight rural counties that have at least 200 HIV cases. We will randomly select 42 of them (stratified by urban vs. rural) as our project sites (2 for pilot-testing; 40 for the actual intervention trial).

### Intervention Components

Our proposed multilevel and multimodal resilience-based intervention program includes: (1) a PLWH component; (2) a family member component; and (3) an HCP component.

#### PLWH Intervention

The primary goal of the PLWH intervention is to assist PLWH in identifying and developing internal and external resilience resources to aid in coping with HIV stigma (including layered stigma), to mitigate the negative impacts of HIV stigma, and to improve their clinical health outcomes. The intervention curriculum will consist of five interactive training sessions (2 h each) with four specific areas of resilience-building: individual assets (self-esteem, emotion regulation, positive future orientation), coping with a chronic health condition (medical adherence, stress reduction, healthy lifestyle, self-care), relationship building (family relationship, provider-patient relationship), and social support (identifying and seeking social support at various socioecological levels). Each session will address one or more of these areas through interactive learning activities (multimedia presentations, role-plays, group discussions, games, personal testimonies). The curriculum will also address empowerment at both individual and family levels across sessions.

#### Family Member Intervention

The format of the family member intervention will be similar to the PLWH intervention but will emphasize providing social support for PLWH's resilience building as well as fostering resilience at the family level. The intervention will consist of five sessions of group activities (2 h each), with each session addressing one or more of the following areas: (1) HIV and ART knowledge [utilizing “undetectable equals untransmissible” [U=U] messaging]; (2) support to strengthen the capacity of PLWH and their family members to adapt to living with HIV; (3) relationship building (family relationships, intimate relationships); (4) emotional and behavioral support for PLWH's adherence to care and treatment, including tailored coping or support strategies to address unique needs of some participants who may be more prone to HIV stigma (e.g., sexual and gender minorities and their family members); and (5) self-care. With appropriate consent from both the PLWH and family members, PLWH will be invited to join with family members for two of the five sessions (i.e., relationship building and adherence support) with the goals of improving the interactions of PLWH with family members and discussing common issues and challenges faced by both parties.

#### HCP Intervention

The HCP intervention will have two primary goals: reducing HCP's stigmatizing attitudes and practices toward PLWH and other social identities such as sexual and gender minorities, sex workers, and injection drug users, and improving the provider-patient relationship. The HCP intervention will consist of four 1.5-h sessions addressing the following topics: (1) universal HIV precautions and occupational safety; (2) layered stigma against PLWH (e.g., manifestations of layered stigma in clinical settings); (3) best practices in building a good provider-patient relationship (e.g., reducing stigmatizing attitudes and behaviors toward PLWH, respecting patients' rights for privacy related to care and disclosure); (4) best practices in providing high quality patient care for PLWH (e.g., increasing skills and comfort in working with PLWH); (5) building a supportive medical environment for better care of PLWH; and (6) skills and confidence in delivering stigma reduction messages to coworkers. The HCP intervention will adapt Popular Opinion Leaders (POL) principles by teaching participants skills for initiating/disseminating stigma reduction messages/practices to colleagues in the workplace (47). The sessions will also be highly interactive with role-plays, facilitator modeling, group discussions, games, testimonies, and presentations from PLWH and medical experts.

## Pilot-Testing

### Pilot-Test the Intervention Curricula

All intervention protocols will be reviewed by our community stakeholders for cultural appropriateness, acceptability, and perceived feasibility. In addition, the intervention curricula will be pilot-tested among 30 PLWH (10 females and 20 males), 30 family members (15 biological family members and 15 family members of choice), and 20 HCPs (8 physicians, 12 nurses or other healthcare professionals). These participants will be recruited from the two clinics (one urban and one rural) that will not participate in the actual intervention trial, and the pilot test will adhere to the same inclusion/exclusion criteria of the actual intervention trial. The main purposes of the pilot test are to assess the comprehensibility of the curriculum; cultural appropriateness of the vignettes, materials, and activities for the target population; and logistics of the implementation (e.g., timing of each session) in order to further improve the program's feasibility and acceptability. Based on the results of the pilot testing, a workshop will be held with the intervention facilitators and the US-China research team to finalize changes to the curricula for incorporation into the facilitator manuals and implementation protocols.

### Pilot-Test the Assessment Instruments

Most of the demographic, psychosocial, and behavioral measurements we have selected for PLWH, family members, and HCPs in this study were field-tested and validated previously in China and have been shown to be reliable and valid for their stated purposes. The measures will be further modified based on the specific aims of this study and global literature on stigma and resilience research among PLWH (21, 22, 48–51). The final drafts of all measures will be reviewed and pilot tested among 40 PLWH, 40 family members, and 20 HCPs to obtain perspectives from the target populations on the clarity, cultural sensitivity, and appropriateness of relevant measures. Pilot testing will follow the same recruitment and consenting process as the actual intervention trial.

## Intervention Trial

### Eligibility Criteria

#### PLWH

The eligibility criteria for PLWH include: (1) aged 18 years or older; (2) at least 3 months since confirmed diagnosis of HIV and/or AIDS; (3) detectable viral load (e.g., viral load ≥50 copies/mL) or a viral round during the past year (i.e., a confirmed detectable viral load following a suppression); (4) willing to refer or give permission for us to contact one of their adult family members (either of origin or of choice) to participate (but the decision to participate will solely reside in the family member); (5) willing to provide a hair sample for testing hair cortisol, hair antiretroviral (ARV) concentration, and relevant biomarkers; (6) willing to consent for the retrieval of past viral load and CD4 count data from their medical records; and (7) willing to be randomized to receive the intervention at different time points in the stepped wedge trial. The exclusion criteria for PLWH include (1) cognitive or physical inability to respond to assessment questions or to participate in intervention; (2) currently incarcerated or institutionalized for drug use or sex work; (3) participating in other intervention activities during the current study period; and (4) plan to permanently relocate outside of the province within a year. Mental and physical inability will be screened by the local research team in consultation with physicians at the participating clinics.

#### Family Members

The eligibility criteria for family members include (1) aged 18 years or older; (2) either family member of origin or family member of choice who provides emotional and other social support to PLWH enrolled in the study; and (3) willing to be randomized (along with PLWH enrolled in the study) to receive intervention at different time points. The exclusion criteria for family members will be the same as for PLWH. The decision of a family member to or not to participate will not affect the eligibility of PLWH.

#### HCPs

The eligibility criteria for HCPs include: (1) age 18 years or older; (2) provides healthcare services at a participating HIV clinic; and (3) has regular contact with HIV patients. The exclusion criteria for healthcare providers include a plan to permanently relocate outside of the province within a year.

### Participants and Recruitment

Participants in the intervention trial will include 800 PLWH and their family members (one per PLWH, either family of origin or family of choice) and 320 HCPs from public HIV clinics in the 40 urban districts/rural townships. Based on our previous work in China, we anticipate high participation rates (e.g., 90% for PLWH, 80% for family members, and 95% for HCPs).

#### PLWH Recruitment

The PLWH sample will be randomly recruited from the 40 participating HIV clinics (~20 PLWH per clinic). A systematic random sampling procedure (52) will be used to randomly select prospective participants from the HIV patient registries. Medical staff or case managers at the clinics will use an arbitrary number (e.g., the date of the month) to identify the first case to be sampled from the list of HIV patients, and then every nth case will be selected where n is a pre-calculated interval for each sampling iteration (e.g., ratio of target sample size to the total # of patients in the pool). The process will be repeated (without replacement) until the target sample size is achieved at each clinic. Once a prospective patient is randomly selected, the local team members (who will visit each clinic at least twice a week during the recruitment period) will verify and confirm their eligibility and schedule a meeting to explain the study design following a standardized recruitment script.

#### Family Member Recruitment

Each PLWH who is eligible and willing to participate in the study will be asked to refer one family member (either of origin or of choice) who is aware of the PLWH's HIV status to participate in the study. The PLWH can give the name to the local research team or bring their selected family member to the clinic for eligibility screening. If the family member is interested, a local study team member will meet with the family member, explain the study design, and obtain appropriate consent. With appropriate consent, we will collect brief information (e.g., family structure, risk profile, and treatment profiles) for those PLWH who either cannot identify a family member (of origin or choice) or whose family member refuses to participate for potential secondary analysis to inform the refinement and scale-up of the proposed intervention in the future.

#### HCP Recruitment

We will randomly recruit 320 HCPs from the 40 participating clinics (~8 per clinic) where we recruit PLWH. Each of these HIV clinics is typically staffed with 15–20 HCPs, including physicians, nurses, case managers, counselors, and medical social workers who have regular contact with HIV patients. With the permission from site leaders, we will stratify the HCPs in a clinic by profession (physicians vs. other) and professional rank (high/middle vs. low) and randomly approach providers in each stratum, explain the study design following a standard script, and invite them to participate. Local team will meet with interested providers (along with site leaders if desired by providers) to answer questions related to schedule, site support, and other logistic issues related to their participation in the study.

## Randomization and Intervention Delivery

We will use a stepped wedge cluster randomized trial for intervention assignment and delivery (Table 1). For logistic reasons, the 40 clinics will be grouped into 5 clusters (8 clinics in each cluster) in each urban/rural stratum based on the geographic proximity. The local research team will randomly assign each cluster to one of the 5 intervention starting dates (e.g., Month 19, 25, 31, 37, or 43) using a single sequence of random assignments (e.g., using a shuffled deck of five cards bearing numbers from 1 to 5). The PLWH and family members will be organized into small (and separate) groups (~10 participants per group) in each clinic through which to receive intervention sessions.

**Table 1:** Stepped wedge cluster randomized trial design.

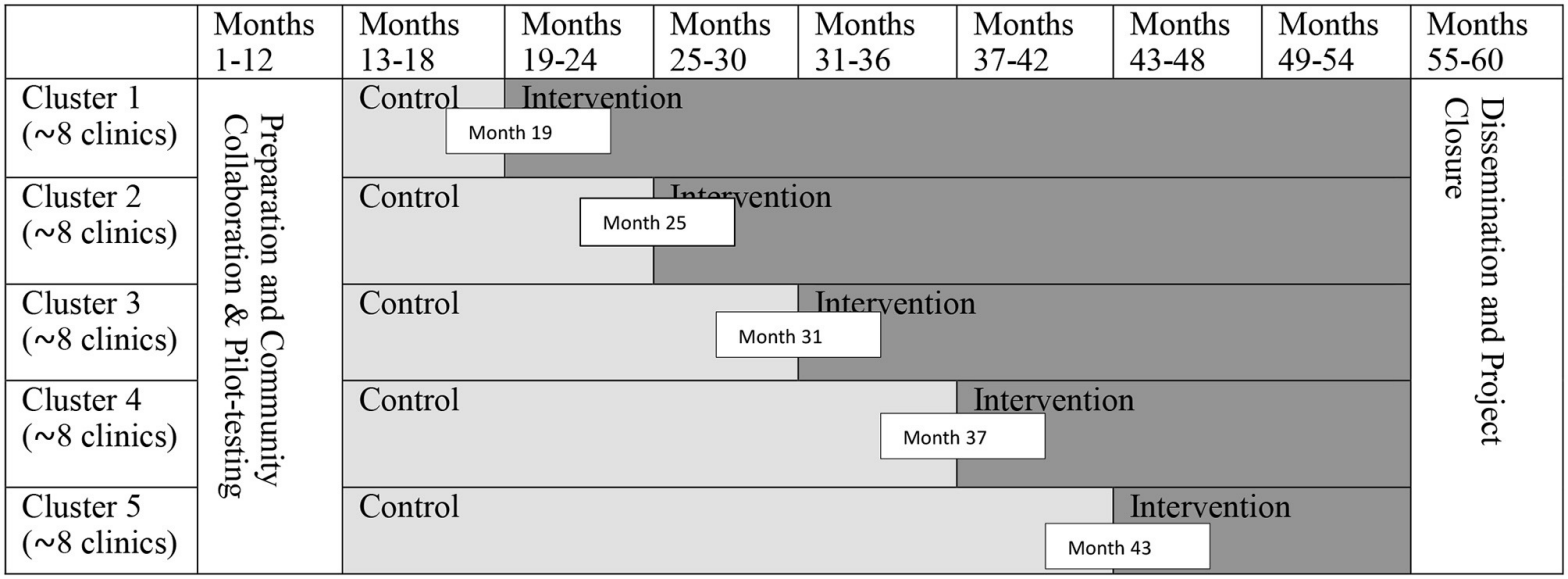

### PLWH Sessions Delivery

The five 2-h PLWH intervention sessions will be delivered over 5 weeks (one session per week) in the clinics where the PLWH are recruited or in nearby private spaces in community centers. Two trained facilitators will deliver the materials through interactive trainings that include multimedia presentations, group discussions, role-play, personal testimonies, and games. The same two facilitators will deliver all five sessions within a clinic to increase group cohesion and rapport with PLWH. The day and time for sessions will be scheduled in advance, and all participating PLWH will be able to opt in to receive a voice or text reminder from the facilitator on the day before the scheduled session.

### Family Member Sessions Delivery

The intervention sessions for family members will be similar to PLWH sessions in terms of format and content and will be led by trained facilitators. Family member intervention sessions will emphasize supporting PLWH to cope with HIV-related stigma and to assist them in improving their clinical outcomes. The majority of sessions will consist of only family members, but PLWH will join their family members for two specific sessions (relationship building and adherence support). To improve the participation of family members, both PLWH and family member sessions will be scheduled on the same days in the same location (so they can travel together). Both participating PLWH and their family members will be consulted during the consenting process on the optimal location and schedules of these group sessions. Family members will also be able to opt in to receive voice or text reminders in advance of scheduled sessions.

### HCP Sessions Delivery

The HCP intervention curriculum consists of four 1.5-h sessions that will be delivered in small groups in the clinic-setting by trained facilitators (i.e., health educators from Guangxi CDC). Ideally, all of the participating HCPs in a clinic will attend the sessions together. However, given the variation of clinical schedules among HCPs in a primary care setting, the delivery schedule and format will be flexible and individually tailored (e.g., four sessions can be given at a rate of one per week or consolidated into two longer sessions). Immediately following randomization, the two intervention facilitators (paired for each intervention delivery) will review scheduling and administrative aspects of the clinics and contextualize each session as necessary based on the providers' workload and clinic schedule. Facilitators will also work with providers during the consent process to develop an individualized plan/schedule in each clinic for assessment, intervention, and follow-up. All HCPs will be able to opt in to receive voice or text reminders for planned sessions.

### Program Fidelity

The proposed interventions in this study are intensive and complex, which is necessary to create and sustain meaningful changes in participants. While challenging, adherence or fidelity to the content and delivery of the program across clusters is also critical. In addition to a standard implementation protocol, which includes the uniform training and certification of the intervention facilitators, we propose the following two steps to assure fidelity to content and delivery of the intervention protocol and to assess factors that influence the implementation of the protocol: (1) Monitoring the actual intervention implementation. Following the established procedures implemented in China (53–61), one of the intervention facilitators will complete a Fidelity Process Form (FPF) for each session. The FPF will contain the essential elements and key process measures of the intervention sessions, including content delivered, time allocated, participation rate, and main activities covered by the sessions. The local team staff will collect and analyze the FPF promptly. If any discrepancy between the protocol and the actual implementation emerges, the intervention facilitators will be informed in a timely manner and necessary steps will be taken to prevent deviations from the intervention protocol; and (2) Audio-recording all sessions. The research staff will randomly select and listen to audio recordings of 20% of the sessions and will then complete an FPF for these recorded sessions. These “independent” process measures will be compared with the ones completed by the facilitators, and feedback concerning fidelity will be provided to the facilitators promptly. Data on the fidelity deviation (e.g., dose variation or substantial modification of activities) will be documented as part of the process evaluation to explore the effect of such fidelity deviation on intervention outcomes.

## Data Collection Procedures

### PLWH and Family Member Surveys

Interviewers (who will be blinded to the schedule of intervention delivery) will administer the baseline and all follow-up surveys to participants *via* tablet computers. The tablet will display and read (with a real human voice, utilizing a headset) the survey questionnaire in a private room (e.g., doctor's office) at district/township hospitals where the participants are recruited. This method will help to ensure the privacy and quality of the data collection, and it will also help to reduce the potential impact that varying degrees of literacy have on individual's ability to understand the items. Clarifications or assistance (with the tablet) will be provided on site by the interviewers as needed. It is estimated that the survey will take about 60–90 min to complete. Participants will be instructed to take a short break after every 30 min of assessment or as needed.

### HCP Survey

All participating HCPs will complete baseline and follow-up surveys. The questionnaires will be self-administered *via* a tablet, and interviewers will be present during the survey to provide necessary clarification. It is estimated that the HCP survey will take about 30 min to complete.

### Hair Sample Collection

The collection of hair samples from all consenting PLWH at baseline and each annual follow-up will follow the Society of Hair Testing guidelines for drug testing in hair (62). Specifically, 1-cm hair sample (20–30 strands of hair for each sample) will be cut as close to the scalp as possible from the vertex posterior region. The hair thatch then will be completely enclosed by a piece of foil and be sealed into an individual plastic bag labeled with the participant ID. Each hair collection process takes about 2 min. All hair samples will be stored at room temperature prior to shipment to the laboratory for process and assays. Laboratory assays using liquid chromatography/tandem mass spectrometry (LC/MS/MS) methods have been developed and validated to analyze most commonly prescribed ARVs in China such as lamivudine (3TC), lopinavir (LPV), ritonavir (RTV), zidovudine (AZT), nevirapine (NVP), efavirenz (EFV), or tenofovir (TFV) in hair samples (63, 64).

## Key Study Variables

***Primary measures* for PLWH** will include: (1) HIV clinical indicators (viral load, CD4 count, and disease progression using the HIV staging system by WHO) to be collected from their medical records with consent; (2) HIV-related stigma, including perceived (or anticipated) stigma, internalized stigma, enacted stigma (65–68), as well as layered stigma against other social identities (e.g., sexual and gender minority, sex work, drug use, migratory status, or poverty); (3) Resilience-related measures of PLWH [coping strategies (69, 70), positive future expectation (71), hopefulness for the future, perceived control over the future (71), personal resilience strengths (72), self-concept (73), relationship self-efficacy (74), emotional regulation (75), self-esteem (76)], family support (perceived emotional support and adherence support, quality of relationship) (77), community support [perceived social support (78)], and healthcare system-related support (perceived acceptance and trust from healthcare facilities and HCPs, quality of provider-patient relationship).

***Intermediate measures for PLWH and family members***will include (1) stress and mental health [self-report measures of depressive symptoms (CES-D) (79, 80), anxiety (81), and stress [Perceived Stress Scale] (82), and stress-related biomarkers [i.e., hair cortisol]]; and (2) adherence to clinical appointments and medication [both self-report (83, 84) and hair ARV concentration for PLWH who are on ART for at least 4 weeks]. In addition, both PLWH and their family members will complete measures of substance use [tobacco use, alcohol use (AUDIT) (85), and other drug use], sexual behavior and reproductive health, HIV-related quality of life (MOS-HIV) (86), and HIV disclosures (77, 87).

***Individual and contextual characteristics (potential moderators)***. Measures we have used in previous studies will also be utilized in this study to collect information from PLWH and their family members on individual and contextual characteristics that may potentially moderate the effect of intervention. These measures may include socioeconomic status, family composition, HIV diagnosis history and treatment history, co-infection, HIV-infection among partners or other family members, and experience of treatment side effects (for those PLWH on ART).

***Measures for HCPs at both the individual level and institutional level***. The individual level measures to be completed by HCPs will include: (1) attitudes and behaviors toward PLWH; (2) attitudes toward privacy or confidentiality protection (88); (3) comfort and self-efficacy in supporting PLWH in their treatment and adherence (88); (4) perceptions of patients' rights to HIV testing and disclosure; (5) perceived provider-patient relationship; (6) knowledge and practice of universal precautions (31); and (7) provider mental health [e.g., depressive symptoms (CES-D) (79, 80), burnout]. The institutional level measures will include both HCP's perceptions and actual observations of facilities' efforts and environments (e.g., policy, guidelines for clinical practice) to integrate stigma reduction into facility culture and clinical practice (89, 90). These measures may include: (1) presence of leaders/team of stigma reduction “champions;” (2) presence of code of conduct and patient “rights;” (3) presence of anti-stigma posters; and (4) presence of additional supportive resources for PLWH and their families.

## Power Analysis

Because of the absence of empirical data on the effect of resilience-based stigma reduction and mitigation intervention in China or any other LMICs, we will conservatively assume a “smaller-than-medium” effect size (Cohen d = 0.35) for the long-term effect (i.e., 36-month follow-up) of our proposed intervention on the primary PLWH outcomes [e.g., CD4 and viral load in terms of continuous measures (log transformation) and categorical measures (e.g., <500 vs. ≥500 for CD4; detectable vs. undetectable for viral load)]. According to the sample size estimation procedure developed for conventional parallel cluster trials by Cohen (91), 140 participants are needed in each cell (or a total 280) to detect an effect size of 0.35 (Cohen d) in various PLWH outcomes at 36-month follow-up with a two-tailed test at alpha = 0.05 and a power of 0.80. However, since the unit of randomization in the current study is the clinic, the sample size calculation, assuming that the unit is the individual, needs to be adjusted for clustering and design effects. Woertman et al. developed a formula to adjust for the design effects of both clustering and the stepped wedge design (DEsw) (92): DEsw = (1+ρ(ktn+bn-1))/(1+ρ(1/2 ktn+bn-1))·(3(1-ρ))/(2t(k-1/k)); where ρ is the ICC, k is the # of steps, t is the # of measurements after each step, n=#of individuals per cluster; and b is the # of baseline assessments. Because no clinic level ρ is available in the literature, we conservatively assume a large ρ = 0.10 for clustering effect (for 2 groups in each of the 40 clinics with ~10 PLWH per group). According to Woertman's procedure (92), a sample size of 528 is needed to produce an effect sample size of 280 with an ρ=0.10. Based on an assumed 10% annual attrition, n= 800 at baseline will produce a sample size of 583 by the 36-months follow-up. Thus, our sample of 800 at baseline (or 583 at 36 months follow-up) will provide adequate power to test the main hypotheses in this study.

## Data Analysis

The stepped wedge design offers some analytic advantages in assessing the intervention effects: (1) The intervention effects can be estimated from both between- and within-cluster comparisons with clusters acting as their own controls; and (2) clusters will receive the intervention at varying time points, providing more flexibility in modeling the effects of intervention timing or consistency in effect across clusters (93).

### Testing Primary Hypotheses

The evaluation of the short-, medium-, and long-term efficacy of the proposed intervention will be based on the “intent-to-treat” model. Following the recommendations by Hussey and Hughes (93), the hypothesized intervention effect on PLWH, family member, and HCP outcomes will be assessed using multivariable statistical methods for data from stepped wedge design, including Mixed Effect Model (SAS procedure PROC MIXED) for continuous outcome measures and the Generalized Linear Mixed Effect Model (PROC GLIMMIX) for categorical outcome measures. The mixed effect analysis approach is advantageous for assessing the intervention's effect since it accounts for a number of key factors, including hierarchical data structure, intraclass correlation (ICC) due to cluster randomization, potential baseline differences in the outcome measures, correlated data due to repeated measures over time, and missing data due to attrition (94–96). In conducting these model-based analyses, exposed (intervention) and unexposed (control) observation periods in the stepped wedge design will take the place of “arms” (i.e., intervention assignment) in conventional parallel cluster trials (93). A significant interaction between the intervention and time will be used as the evidence of an intervention effect. The stepped wedge design will also allow us to examine the way in which the intervention effect develops over time once it is introduced into a cluster and allow us to explore the variation of intervention effects among clusters, using within-cluster comparison of unexposed and exposed observations (93).

### Mediation and Moderation Analyses

Mediation will be tested using the methodology developed by Baron and Kenny (97). The method is based on multiple regression models focusing on the predictor (e.g., intervention status), the mediator (e.g., improvement in intermediate outcomes), and the primary intervention outcome. According to Baron and Kenny (97), a mediation effect is identified in line with three conditions: (1) the predictor is significantly associated with the outcome; (2) the predictor is significantly associated with the mediator; and (3) the effect of the predictor on the outcome is significantly reduced in magnitude after including the mediator in the model. The PROC MIXED and PROC GLIMMIX procedures will be used to perform these analyses with continuous and categorical data, respectively. In addition to regression analyses (i.e., PROC MIXED and PROC GLIMMIX), the Sobel test, as specified by MacKinnon and Dwyer (98), will be used to determine the significance of the mediation effect. To test the moderation effect of contextual factors, we will also follow the procedure suggested by Barron and Kenny (97). We will employ multiple regression analyses and test the significance of the appropriate interaction terms between intervention status and the potential moderators (e.g., PLWH's gender, sexual identity, migratory status, family structure, risk profile, or disease stage) so that the future scale-ups of the intervention can be appropriately informed. Structural equation modeling (SEM) using LISREL will be employed as a complementary methodology to conduct mediation and moderation analyses (99, 100).

## Discussion

The limited success of existing HIV stigma reduction efforts requires new approaches that move beyond existing practice (8, 9, 13, 101). This study applies an innovative stepped wedge cluster randomized trial, provides an alternative to an “eradication-only” approach, and broadens our conceptualization to include both reduction of stigma and development of resilience. The proposed intervention focuses on the strengths, competencies, resources, and capacities in PLWH, their families, and HCPs in not only reducing HIV-related stigma but also actively mitigating the negative effects of stigma. The proposed study will develop a culturally appropriate, effective, and sustainable intervention modality that extends beyond the level of individual PLWH to include interpersonal, structural, and social determinants of HIV-related stigma. The resilience-based approach focusing on building the strengths of PLWH and their key social support systems is particularly appropriate within the cultural context of China and other LMICs where PLWH and their family members are often strongly marginalized and discriminated and often face layered stigma which poses significant challenges to behavioral interventions that aim to eliminate or eradicate such layered stigma (88, 102–107). Informed by both positive psychology and socio-ecological theory, our conceptualization of a resilience-based intervention is a critical step in both understanding resilience within the multiple contexts of PLWH, their families, and their HCPs and improving clinical and other health-related outcomes of PLWH.

The RISE-UP intervention will help PLWH to enhance their personal strengths by providing skills-based training in adaptive coping, problem-solving, relationship building, and social support utilization in a group setting that fosters peer social support. The building of internal strengths among PLWH will be further supported and strengthened by efforts that engage family members and HCPs. According to resilience theory, these two key social support systems are critical for successful adaptation to adversity; the internal strengths and the presence of strong support systems from family members and healthcare facilities will improve PLWH's psychological health (e.g., decreased symptoms of anxiety and depression, reduced stress, improved future orientation) and will increase PLWH's engagement in HIV care and adherence to ART; and the improvement in psychological health and treatment adherence will further improve PLWH's immune functioning, which in turn will improve PLWH's viral suppression and other clinical outcomes (e.g., CD4 count, quality of life) and prevent disease progression, as well as prevent spread of HIV to others (U=U) and end the HIV epidemic.

One of the challenges associated with conducting longitudinal studies is attrition. Effective strategies that engage and retain cohort participants are critical to the integrity of research outcomes. Significant and systematic attrition can reduce the generalizability of outcomes and the statistical power to detect effects of interest. The effective retention strategies in this study will include: (1) An individualized follow-up plan. Because of the diversity in PLWH's living situations and daily schedules, we will advise the participants during the consenting process that they will be followed over time and will consult with each participant about the best way to maintain contact. In this way, we will develop an individualized, flexible follow-up plan for each participant (e.g., optimal way to contact them, optimal time and place to meet, preferred frequency of contacts between assessments); (2) Providing all PLWH and family members with the local team member's cellphone number for them to notify research staff of any address change. In addition, we will ask for at least three alternative contacts (i.e., relatives, friends) who can provide information on the whereabouts of the participant in case of relocation; and (3) Arranging telephone or online contact or home visitation prior to each follow-up assessment and making at least three attempts at contact if a PLWH or family member cannot be located. For confidentiality reasons, none of the communications to alternative contacts will reveal the nature of the study.

Guided by the conceptual framework, we hope that the proposed multilevel and multimodal resilience-based intervention can mitigate the negative effects of HIV-related stigma on the clinical outcomes of PLWH. The proposed program is informed and supported by the extant global literature on HIV-related stigma reduction, resilience research, and our preliminary data. Findings from this rigorously designed study are anticipated to inform future interventions and evidence-based policymaking by identifying strategies to build on the strengths of PLWH and their socio-ecological systems in resource-limited settings.

## Study Dissemination

To materialize the anticipated social benefits of the proposed research and to maximize the impact of the proposed resilience-based intervention, we will take the following strategies to disseminate the study findings. First, local community forums. We will hold meetings with our community advisory board, local partners and other key stakeholders (including the PLWH and their family members and healthcare providers) in each project site (urban districts or rural townships) to present study findings and prepare a strategic dissemination or scale-up plan with local communities, healthcare systems, NGOs, and government agencies if applicable; Second, scientific communities. We will publish the data in international and national scientific journals and present the study findings at national and international scientific meetings and conferences. We will capitalize on social media and professional networks that can increase the reach and accessibility of findings, such as open access publication, webinars, files and videos available on websites and publicly available channels (e.g., YouTube), to increase visibility and impact of the scientific publications and presentations; Third, impact on policy. The dissemination efforts of this project will extend beyond the scientific arena and will also target policy makers in China at local (participating sites), regional (Guangxi) and national levels through various policy forums, policy papers, and special presentations. We hope that the anticipated success of the proposed project will prompt policy changes in HIV prevention, treatment and care in China and the lessons learned from the project and the tested intervention strategies (curricula, delivery, and evaluation) can be scaled-up to improve health outcomes of PLWH in China and other LMICs.

## Ethics Statement

This study protocol was reviewed and approved by the University of South Carolina Institutional Review Board (Protocol# Pro00099388) and the Guangxi Institutional Review Board (Protocol# GXIRB2020-39-1). The patients/participants will provide their written informed consent to participate in this study.

## Author Contributions

XL is the principal investigator of this project and led the study design. SQ contributed to the development of the study. XY and XL led the writing of this protocol manuscript. SH and CT contributed significantly to the editing of this manuscript. ZS and YZ contributed to the conception and design of the study. All authors reviewed and provided comments to improve the manuscript, contributed to the editing, and final approval of the protocol.

## Funding

Research reported in this publication was supported by the National Institute of Mental Health of the National Institutes of Health under Award Number R01MH127961. The content is solely the responsibility of the authors and does not necessarily represent the official views of the National Institutes of Health.

## Conflict of Interest

The authors declare that the research was conducted in the absence of any commercial or financial relationships that could be construed as a potential conflict of interest.

## Publisher's Note

All claims expressed in this article are solely those of the authors and do not necessarily represent those of their affiliated organizations, or those of the publisher, the editors and the reviewers. Any product that may be evaluated in this article, or claim that may be made by its manufacturer, is not guaranteed or endorsed by the publisher.

## Abbreviations

AIDS: Acquired Immunodeficiency Syndrome
ART: Antiretroviral Therapy
ARV: Antiretroviral
AZT: Zidovudine
CDC: Centers for Disease Control and Prevention
EFV: Efavirenz
HCP: HealthCare Providers
LPV: Lopinavir
LMICs: Low- and Middle-Income Countries
MSM: Men Who Have Sex With Men
NVP: Nevirapine
PLWH: People Living With HIV
POL: Popular Opinion Leaders
RTV: Ritonavir
SEM: Structural equation modeling
TFV: Tenofovir
3TC: Lamivudine.
